# Bayesian Methods: A Means of Improving Statistical Power in Preclinical Neurotrauma?

**DOI:** 10.1089/neur.2024.0028

**Published:** 2024-07-16

**Authors:** Peyton M. Mueller, Abel Torres-Espín, Cole Vonder Haar

**Affiliations:** ^1^ Department of Neuroscience, Injury and Recovery Laboratory, Ohio State University, Columbus, Ohio, USA.; ^2^ School of Public Health Sciences, University of Waterloo, Waterloo, Canada.

**Keywords:** data analysis, hypothesis testing, statistics

## Abstract

The field of neurotrauma is grappling with the effects of the recently identified replication crisis. As such, care must be taken to identify and perform the most appropriate statistical analyses. This will prevent misuse of research resources and ensure that conclusions are reasonable and within the scope of the data. We anticipate that Bayesian statistical methods will see increasing use in the coming years. Bayesian methods integrate prior beliefs (or prior data) into a statistical model to merge historical information and current experimental data. These methods may improve the ability to detect differences between experimental groups (i.e., statistical power) when used appropriately. However, researchers need to be aware of the strengths and limitations of such approaches if they are to implement or evaluate these analyses. Ultimately, an approach using Bayesian methodologies may have substantial benefits to statistical power, but caution needs to be taken when identifying and defining prior beliefs.

## Introduction

Worldwide, traumatic brain injury (TBI) affects up to 69 million people annually,^1^ and 250,000–500,000 people incur a spinal cord injury (SCI).^2^ These injuries can generate heterogeneous outcomes from central nervous system damage. Symptoms can present neurologically as chronic pain, paresis and paralysis, or autonomic dysfunction^3–7^; psychologically as increased anxiety, depression, and cognitive impairments^4,5,8–10^; or developmentally as an increased risk for neurodegenerative diseases.^11,12^

To better understand how injury pathology is related to symptoms and develop potential therapies, preclinical models are necessary. These animal models provide experimentally controlled settings that can be impossible to achieve in clinical populations, typically allow for collection of a greater amount of longitudinal data from multiple assessments, and can enable detailed postmortem pathological measures.^13–15^ Despite the benefits of preclinical models, there are both ethical (e.g., animal lives) and practical (e.g., animal housing, veterinary care, personnel) costs and physical limits on research throughput. The recent National Institutes of Health (NIH)-wide emphasis on rigor and reproducibility in research has many fields considering how to best power and optimize studies for reliable and replicable effects, but individual laboratories must balance this against increasing costs of research. Thus, an important question for biomedical laboratories is how to appropriately determine the number of animals needed in a preclinical study to preserve power while minimizing use of animal lives and research costs.

Sample size estimation is often approached from a statistical lens, using methods such as pre-experiment power calculation.^16,17^ However, these methods do not take into consideration the practical issues named earlier. Preclinical experiments may fall far below the necessary sample for well-powered experiments,^17^ which will reduce the precision of the measured effect. The consequences of small sample sizes in a research study can be costly for the individual laboratories and the research community.^17,18^ For instance, an important finding may be missed (thus wasting time/money/lives on other avenues), or researchers might overinterpret marginal or spurious findings to try and salvage something publishable (thus wasting time/money/lives on expansion and replication).^18,19^ Next, we outline statistical considerations for these issues and discuss the increasing relevance of understanding Bayesian analyses for the average researcher.

## Statistical Power, Inferential Errors, and Power Analysis

Statistical power is the ability to detect an effect when it is present or a true effect (e.g., group differences).^17,18^ In hypothesis testing, unavoidable inferential errors may occur—type I (false positive) and type II (false negative) errors.^17^ These are typically held in balance by a combination of decisions about statistical test thresholds and experimental design. Under perfect conditions (i.e., sufficient sample size and appropriate statistical test), the probability of a false positive is equal to the α-level (i.e., significance threshold level) and is set before experimentation. In most cases, the α-level is kept low (commonly 0.05) to reduce the likelihood of encountering a false positive.^18^ However, a low α-level also decreases the ability to detect differences or statistical power.^17,18^ The probability of encountering a false negative is the β-level and is often set at two to four times the α-level. The mathematical definition for power in a statistical test is 1-β (where 1 is the highest possible probability of detecting an effect).^17,18^

Statistical power is also affected by other factors, which can be controlled experimentally.^16,17^ Sample size influences power such that the larger the sample size (*n*), the higher the precision of an analysis—hence, higher statistical power.^16–18^ Statistical theory states that the magnitude of an experimental effect/manipulation also influences the ability to detect it. The larger the effect size, the smaller the sample size needed to detect it. However, effect size may be difficult to manipulate experimentally while replicating real-world effects and under logistically challenging studies for preclinical neurotrauma. The relationships between α, β, effect size, and *n* can be used to calculate and inform experimental design or statistical analysis, a process known as power analysis.^16–18^ Researchers may conduct *a priori* power analyses to determine the *n* required to detect a specific effect size given α and β values or a *sensitivity* power analysis to determine what effect size could be detected with a given *n*, α, and β when the true effect size is unknown (e.g., with a novel therapy).^16,18^ This process is aided by various tools such as G*Power.^20^

While the above presents scientific practice under ideal conditions, this is rarely the reality for researchers. As previously discussed, the monetary costs and logistical complexity associated with animal research are substantial, and this, combined with ethical imperatives, pushes scientists to use as few subjects as possible.^16^ However, if they wish to conserve sufficient statistical power to detect true treatment effects, they must have as many subjects as possible.^17^ Given this obstacle, an update from traditional statistical methodology applied by most biomedical researchers to more modern approaches may be needed.

## The Bayesian Approach

**FIG. 1.**
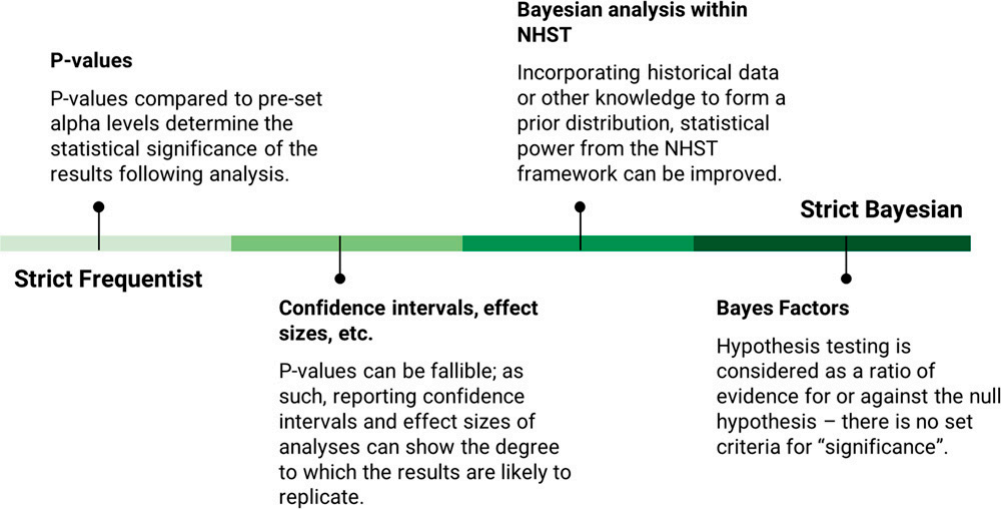
A diagram emphasizing that most statistical approaches exist on a continuum between strict Frequentist and Bayesian interpretations. Blending approaches allow scientists to integrate the strengths of each in their research.

Thus far, we have focused on the null hypothesis significance testing (NHST) or Frequentist framework taught in many introductory statistics courses in the biological sciences. An alternate view of statistical testing comes from the Bayesian perspective.^21^ Although NHST requires some criterion level (i.e., α) to determine whether the results of a test are statistically significant or not, Bayesian analyses recommend against arbitrary cutoffs and instead place evidence on a spectrum.^21,22^ Essentially, empirically observed data provide a degree of evidence either for or against some prior beliefs. Bayes’ theorem can colloquially be described as the degree of belief in an observed event, given one’s knowledge of the conditions surrounding that event (e.g., observed in previous experiments). Thus, the observation of an event that is *known to be highly improbable* (“prior” belief) is likely to be a false positive, but for repeated observations of that event, one might *update their view that it is improbable* (“posterior” belief). Thus, Bayes’ theorem provides the ability to consider current results in the context of prior data.^21,22^ Although we have presented the more extreme ends of Frequentist and Bayesian perspectives, these can be placed on a continuum (Fig. 1). Indeed, many researchers have argued for softening NHST to rely less on the *p* value and focus on effect sizes and confidence intervals, moving NHST toward the Bayesian perspective.^23,24^

**FIG. 2.**
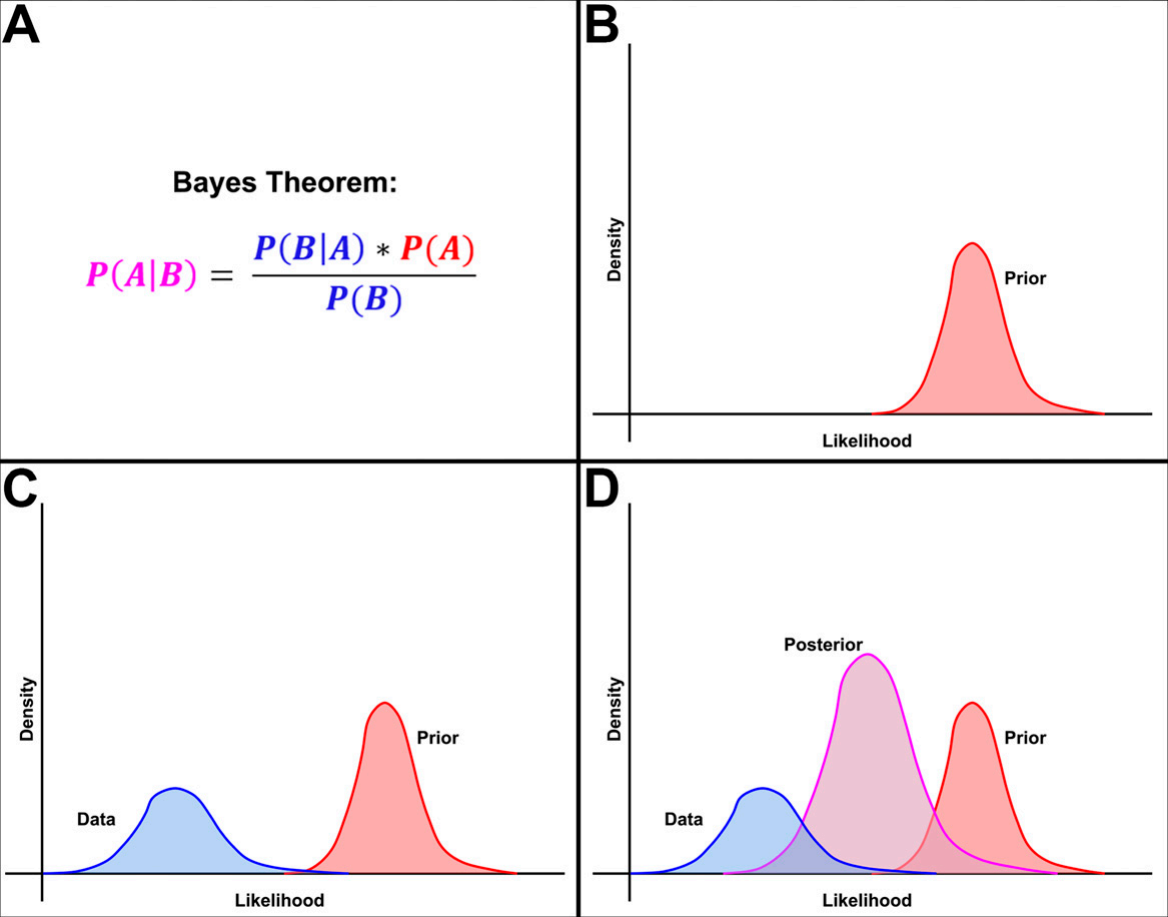
Depiction of how experimental data and prior distributions generate a posterior distribution according to Bayes’ theorem. **(A)** Mathematical expression of Bayes theorem, color coded to distributions in subsequent panels. **(B)** A distribution centered around some previous belief or hypothesis [P(*A*)]. **(C)** Data are empirically collected and plotted [P(*B*|*A*) / P(*B*)]. **(D)** A posterior distribution [P(*A*|*B*)] is generated by multiplying the two distributions according to Bayes’ theorem.

Mathematically, Bayes’ theorem determines the probability of a particular event (or set of data), *A*, given some information about a different event, *B* expressed as P(*A*|*B*), known as the conditional probability of *A* given *B*. This is equal to the probability of event *B* occurring given event *A* occurred (*B*|*A*) multiplied by the probability of event *A* occurring, all divided by the probability of event *B* occurring (Fig. 2A). In Bayesian statistical analysis, P(*A*), the degree of belief in *A* occurring (sans information about event *B*) is estimated as a *prior distribution* (Fig. 2B), and in the case of an experiment, it captures our belief or previous knowledge about the potential results. The empirical data are represented as the distribution of the remaining fraction P(*B*|*A*) divided by P(*B*) (Fig. 2B). Multiplying these two together forms the *posterior distribution*, P(*A*|*B*) (Fig. 2C). This posterior is ultimately the conclusion regarding the results.

Given that the *philosophy* of Bayesian hypothesis testing (strength of evidence for decision) is diametrically opposed to NHST (defined threshold for yes/no decision),^21,22^ individuals may be hesitant to overhaul their framework of how they consider experimental results. However, Bayesian *methodology* may provide a means to improve statistical power (i.e., the probability of detecting a true effect) under an NHST framework by incorporating historical data or other prior beliefs.^25,26^ Rather than using a strict Bayesian approach in which results only provide evidence for or against the initial hypothesis, one can set a criterion for “statistical significance.” For instance, Bayesian methods can generate a *credible interval*, a range indicating the probability of a measure of interest falling within it.^27^ To compare with an α of 0.05, researchers might use a 95% credible interval (5% chance of range *not* containing measure of interest). With this credible interval, if the measure of interest does *not* contain the null hypothesis value, the effect could be considered “statistically significant.” This uses the strength of Bayesian methodology without researchers having to completely abandon the NHST framework with which they are familiar.

Although this may seem abstract, there are real-world benefits to implementing Bayesian methodology to improve statistical power. A recent study used simulations from neuroscience data to demonstrate that incorporating historical data into priors improved power in a simple *t*-test.^26^ We recently published a similar demonstration using data simulated from rat TBI datasets containing multivariate data.^25^ In this study, we found that a type of multivariate data (i.e., interdependent data—containing proportions summing to 1) can have excessive rates of false positives when analyzed with general linear models (e.g., analysis of variance). While adjusting statistical test methodology reduced false-positive rates to the target 5%, it also substantially reduced power at typical behavioral neuroscience sample sizes. However, when Bayesian methods were applied, statistical power was increased and false-positive rates remained low.

## Pros and Cons of Bayesian Analysis

As with any new tool researchers may consider, it is critical to understand both the strengths and weaknesses—statistical methodology is no different. One major advantage of the Bayesian statistical philosophy (updating prior beliefs based on new data) is that, at its core, it is a formalization of the research process itself.^21,22^ Consider the following example: say published literature suggests that TBI in rats will cause seizure ∼5% of the time; however, one researcher exposes a cohort of 20 rats to a TBI and finds that 40% of them experience seizures. The researcher compares this observation with the prior estimate that only 5% of rats experience seizures and concludes that this is highly unusual and potentially an erroneous finding. The researcher then repeats the experiments with a new cohort of rats and 35% experience seizures. These results cause the researcher to revise their confidence in the initial *prior* belief that only 5% of rats experience seizures following TBI and develop a new *posterior* belief that the true rate is considerably higher than 5%. As additional evidence accumulates from repeated testing, the posterior distribution gradually shifts until it more closely reflects reality.

One major consideration when applying Bayesian methodology is how to choose appropriate priors. Priors can be considered on a spectrum from noninformative to conservatively informative, moderately informative, or definitively informative (sometimes this is expressed in a spectrum of “weak” to “strong” prior). In the example just given, if a researcher had data going back from 500 rats on seizure in TBI, their prior belief is likely to be much stronger than if they had only tested one cohort previously. This degree of information is quantified in Bayesian testing via the distribution error term of the prior. For example, a prior that used the mean ± 1 standard error of the mean from 500 subjects as the prior would be more informed than one that inflated the error term to ±5 standard errors of the mean. It would also be more informed than a prior originating from a smaller sample size of 40 rats. Because these priors influence the conclusion of the outcome, it is considered good practice to evaluate the influence of prior selection on statistical conclusions (e.g., via prior predictive checks or evaluating and comparing multiple priors).^28^

Another advantage often touted of Bayesian relative to NHST approaches is removing the dependence on the *p* value.^22^ Under the NHST framework, the difference between observed data and the null hypothesized value must be large enough (at a given sample size) to determine if there is “enough” difference between the two to reach significance. Under strict NHST guidelines, for an alpha level of 0.05, a *p* value of 0.049 indicates a significant difference; however, a *p* value of 0.051 indicates that there are no significant differences between groups. Under a Bayesian analysis, either of these results would merely be reported as the strength of the results.^22,29^ Because of pressure to publish only significant results, these strict decision criteria may result in selective or premature reporting of data.^30^ These problems are widely noted, and based on recent literature, the American Statistical Association recommended removing reliance on significance testing and *p* values and instead suggested focusing on “uncertainty, variability, multiplicity, and replicability” (e.g., confidence intervals, effect sizes).^24^ These ideas are in line with a philosophy somewhere between Frequentist and Bayesian (Fig. 1).

Direct integration of Bayesian methodology into NHST without staunch adherence to Bayesian philosophy may provide benefits to researchers. Bayesian methods can use historical data to define a strong prior belief and increase statistical power without increasing subjects in clinical trials.^31,32^ Used appropriately, this could also help balance the various pressures associated with animal research discussed above. However, poor implementation may actually exacerbate issues of rigor and reproducibility. Without a strong understanding of the limits of Bayesian methods, they may be improperly applied as a catch-all fix for poor statistical power. In particular, great care needs to be taken when defining priors. In previous studies using neuroscience-specific data, we and others only defined priors for the *control group* using historical control animal data.^25,26^ A key assumption is that priors come from the same parent population as the observed data; if this is not true (e.g., strain or sex difference that affects outcome), incorporation of priors may actually produce poorer precision and statistical power.^21,29^

**FIG. 3.**
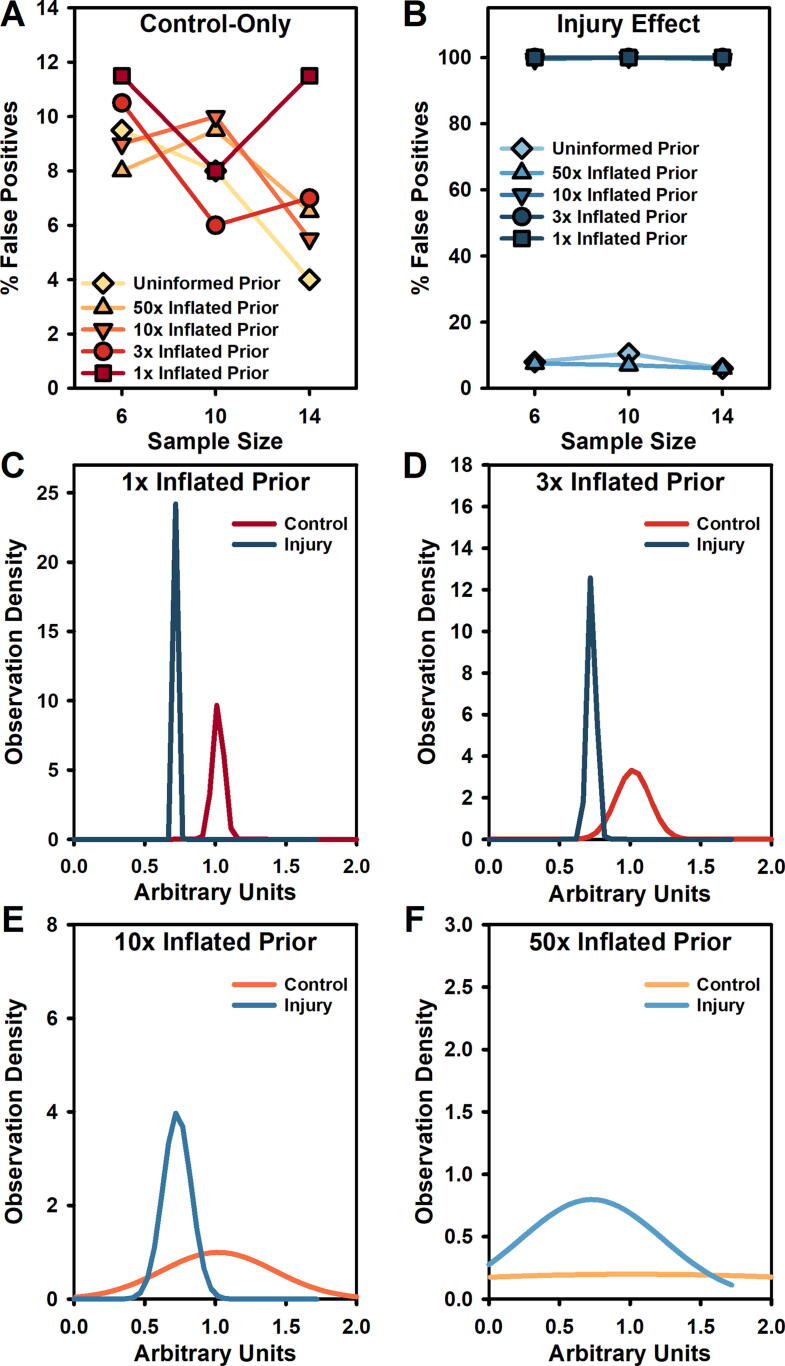
Simulated prior distributions based on the amount of prior information available. The informativeness or strength of the prior is defined from the mean and standard error of historical data, with a 1× inflation corresponding to exactly the historical data (most informative), and each multiplication (3×, 10×, 50×) increasing that error term and reducing the informativeness of the prior. Uninformed priors assume a “flat” distribution or no prior information. **(A)** False-positive rates remain low when only control priors are defined at different levels (ideal target: 5%). **(B)** False-positive rates reached 100% at all but the lowest information priors when the difference between control and injured priors is defined. **(C)** Prior distributions with very informative priors (1×—no inflation) for both control and injury show no overlap. **(D)** Informative priors (3× inflation) show similarly low overlap. **(E)** Even with overlap, medium strength priors (10× inflation) generate a 100% false-positive rate when the difference between control and injury was set as a prior. **(F)** Only with very low-information priors (50× inflation) or flat/uninformed priors (distribution not shown) were false-positive rates reduced to a reasonable level.

While strongly informative priors can increase statistical power, they may also increase false-positive rates for typical preclinical sample sizes.^25^ Our published simulated data show that applying priors to *controls only* improves power while maintaining relatively low false positives.^25^ In this study, we analyze new Bayesian models based on this published set (Fig. 3). When we specify priors for only control animals, false positives remain relatively low across a range of defined prior information (4–12%) and generally decline as the sample size is increased (Fig. 3A). In contrast, if we define prior information specifying the control *and* anticipated injury effects (i.e., the variance of the injury effect), the false-positive rate rises to 100% under all but the least informative priors (Fig. 3B). The compounding effect of multiple, informative prior distributions bias the test toward detecting an effect by specifying the historical difference between control and injured groups, making *not* finding evidence of an effect nearly impossible. This is reflected in the narrow, peaked shape of the prior distribution (Fig. 3C, D). One caveat to these data is that they are likely somewhat exaggerated because of the multivariate and interdependent nature of the data; univariate outcomes may not be as extreme. However, this underscores why we must assess the limits of Bayesian methods.

Integrating historical data in Bayesian analyses is a promising and viable means of improving statistical power. Because the NIH has recently implemented a new set of data principles to ensure that data are findable, accessible, interoperable, and reusable,^33^ a wealth of data will soon be available through repositories such as the Open Data Commons for SCI (odc-sci.org) and TBI (odc-tbi.org).^34,35^ These data can be used to generate prior distributions and applied to experiments to improve the detection of meaningful results without necessarily incurring greater laboratory costs.

## Relevance to the Researcher

If Bayesian methods can truly improve statistical power for preclinical neurotrauma research, as demonstrated in previous literature, why are they not already widely used? We propose that this lack of implementation stems from the following three issues: (1) a lack of statistical education (including continuing education post-PhD) in neuroscience, (2) a reticence for rejecting “tried-and-true” means of analysis, and (3) a lack of easy-to-use generalized tools that allow for these types of analysis. These issues may interact and compound to reduce the prevalence of certain types of analysis in reported literature. A recent study quantified this effect and found that increasing statistical complexity was predictive of lower article acceptance rates.^36^ Similar issues surrounding understanding of statistical power were identified even 20 years ago as impacting publication and grant review.^37^

Up to 70% of neurotrauma articles contain inappropriate statistics or inferences based on the reported analyses.^38^ This may stem from minimal emphasis on statistical education in the life sciences^39,40^; thus, many researchers may be unaware that their analyses are inappropriate or of potential solutions to meet statistical assumptions. This lack of statistical education and continuation of poor analytic procedures in published research both certainly contribute to the replication crisis. This may also limit the likelihood that Bayesian methods (and their limitations) will be effectively incorporated into standard analytic procedures in neuroscience.

In the field of neurotrauma, Bayesian analyses are performed primarily in clinical studies^41,42^; however, the preclinical space is beginning to see adoption of these methods.^43–45^ One concern may be that relatively little is known about the limitations of Bayesian methods for preclinical neurotrauma. Consideration must be given to appropriate definition of prior distribution parameters and how much historical data are necessary or sufficient for inclusion in prior distributions. Thus, the practical benefit to using Bayesian methods over the more familiar NHST may not be immediately apparent, particularly in preclinical experimental settings where more variables may be controlled. However, ultimately, a hybridization approach of Bayesian methods and the NHST framework may have utility for preclinical studies. The Bayesian methods hold great potential for their ability to leverage historical data to detect differences in novel data, but care must be taken in how priors influence interpretation under the NHST framework.

It may be tempting for a researcher who does not plan to implement Bayesian methods to discount the need for understanding them. However, it is likely that these methods will see increasing use in the coming years, even in preclinical studies. Thus, researchers need to be prepared to evaluate the statistical rigor of research reports they consume. This increase may have occurred naturally, but with the new NIH policies on data sharing,^46^ a wealth of historical data will soon be available, which can inform Bayesian priors. Thus, the field of preclinical neurotrauma needs to be prepared to evaluate how and why certain priors are used and whether they lead to appropriate conclusions or bias researchers toward detecting effects.

A solution to the issues of implementing Bayesian methods in preclinical neurotrauma must involve more robust statistical education, both during degree progression and throughout one’s career. To efficiently draw meaningful conclusions from quantitative analysis of research, it may be worth considering some form of statistical continuing education component for researchers, similar to how medical and legal professionals must complete annual continuing education credits. In doing so, researchers would be required to remain up-to-date with the latest and best statistical analytical procedures and will be more likely to use the most appropriate means of analysis (perhaps including the use of Bayesian methods) rather than those with which they are most familiar. In addition, neuroscientists may consider increasing collaborative efforts with trained biostatisticians to improve the precision and efficacy of their analyses and scientific conclusions. The creation of user-friendly programs for researchers to implement Bayesian methods, such as the RePAIR tool,^26^ will also be useful to hasten adoption.

## Abbreviations Used

FAIR: Findable, Accessible, Interoperable, Reusable
NHST: Null hypothesis significance testing
SCI: Spinal Cord Injury
TBI: Traumatic Brain Injury
